# Supplementary material for: High-throughput synapse-resolving two-photon fluorescence microendoscopy for deep-brain volumetric imaging in vivo
Source: eLife. 2019 Jan 4;8:e40805. doi: 10.7554/eLife.40805 (PMC6338462; doi:10.7554/eLife.40805)
Supplement: Transparent reporting form [file elife-40805-transrepform.docx]

***eLife’s* transparent reporting form**

We encourage authors to provide detailed information *within their submission* to facilitate the interpretation and replication of experiments. Authors can upload supporting documentation to indicate the use of appropriate reporting guidelines for health-related research (see [EQUATOR Network](http://www.equator-network.org/%20)), life science research (see the [BioSharing Information Resource](https://biosharing.org/)), or the [ARRIVE guidelines](http://www.plosbiology.org/article/info:doi/10.1371/journal.pbio.1000412) for reporting work involving animal research. Where applicable, authors should refer to any relevant reporting standards documents in this form.

If you have any questions, please consult our Journal Policies and/or contact us: [editorial@elifesciences.org](mailto:editorial@elifesciences.org).

**Sample-size estimation**

- You should state whether an appropriate sample size was computed when the study was being designed
- You should state the statistical method of sample size computation and any required assumptions
- If no explicit power analysis was used, you should describe how you decided what sample (replicate) size (number) to use

Please outline where this information can be found within the submission (e.g., sections or figure legends), or explain why this information doesn’t apply to your submission:

For *in vitro* measurement of lateral point spread function (**Fig. 4**), sample size depends on the number of isolated beads in the acquired image. Data from 6-11 beads were collected and analyzed for each condition, as described in its figure legend.

For *in vivo* functional imaging (**Fig. 10**), sample size was determined by the number of neurons within the field of view. Sessions were carried out in duplicates. Details are described in main text, page 15-16, and the figure legend.

**Replicates**

- You should report how often each experiment was performed
- You should include a definition of biological versus technical replication
- The data obtained should be provided and sufficient information should be provided to indicate the number of independent biological and/or technical replicates
- If you encountered any outliers, you should describe how these were handled
- Criteria for exclusion/inclusion of data should be clearly stated
- High-throughput sequence data should be uploaded before submission, with a private link for reviewers provided (these are available from both GEO and ArrayExpress)

Please outline where this information can be found within the submission (e.g., sections or figure legends), or explain why this information doesn’t apply to your submission:

Details could be found in main text, page 15-16, and legend of **Fig. 10**. Outliers highlighted in the box-whisker plots were included in the statistical analysis.

**Statistical reporting**

- Statistical analysis methods should be described and justified
- Raw data should be presented in figures whenever informative to do so (typically when N per group is less than 10)
- For each experiment, you should identify the statistical tests used, exact values of N, definitions of center, methods of multiple test correction, and dispersion and precision measures (e.g., mean, median, SD, SEM, confidence intervals; and, for the major substantive results, a measure of effect size (e.g., Pearson's r, Cohen's d)
- Report exact p-values wherever possible alongside the summary statistics and 95% confidence intervals. These should be reported for all key questions and not only when the p-value is less than 0.05.

Please outline where this information can be found within the submission (e.g., sections or figure legends), or explain why this information doesn’t apply to your submission:

Statistical analysis method of lateral PSF measurements could be found in the legend of **Fig. 4**.

Statistical details of *in vivo* functional imaging results are described in main text, page 15-16, and the legend of **Fig. 10**.

(For large datasets, or papers with a very large number of statistical tests, you may upload a single table file with tests, Ns, etc., with reference to sections in the manuscript.)

**Group allocation**

- Indicate how samples were allocated into experimental groups (in the case of clinical studies, please specify allocation to treatment method); if randomization was used, please also state if restricted randomization was applied
- Indicate if masking was used during group allocation, data collection and/or data analysis

Please outline where this information can be found within the submission (e.g., sections or figure legends), or explain why this information doesn’t apply to your submission:

Lateral PSF measurements are allocated into two groups (Gaussian and Bessel) to compare their lateral resolutions, as presented in **Fig. 4**.

For *in vivo* functional imaging, data from the same mouse were grouped together. They were further separated into “end of dark cycle” and “end of light cycle” groups and compared. Details are described in main text, page 15-16, and the figure legend of **Fig. 10**.

**Additional data files (“source data”)**

- We encourage you to upload relevant additional data files, such as numerical data that are represented as a graph in a figure, or as a summary table
- Where provided, these should be in the most useful format, and they can be uploaded as “Source data” files linked to a main figure or table
- Include model definition files including the full list of parameters used
- Include code used for data analysis (e.g., R, MatLab)
- Avoid stating that data files are “available upon request”

Please indicate the figures or tables for which source data files have been provided:

Almost all data needed to evaluate the conclusions in the paper are present in the paper or the supplementary materials.
